# Effects of Milk Protein Hydrolysates on Skin Quality and Advanced Glycation End Products in Women With Perceived Skin Dullness: Two 8‐Week Randomized Controlled Trials

**DOI:** 10.1111/jocd.71032

**Published:** 2026-07-10

**Authors:** Sayuri Arai, Naoki Yuda, Masaki Kurimoto, Ryota Suzuki, Hajime Nakada, Hiroshi Ochi, Miyuki Tanaka, Atsushi Nakajima, Yuki Yamamoto, Masatoshi Jinnin

**Affiliations:** ^1^ Food Function Research Institute, R&D Division Morinaga Milk Industry co. Ltd. Kanagawa Japan; ^2^ Ueno‐Asagao Clinic Tokyo Japan; ^3^ Department of Dermatology Wakayama Medical University Wakayama Japan

**Keywords:** advanced glycation end products, casein hydrolysate, randomized controlled trial, skin aging

## Abstract

**Background:**

Milk protein hydrolysates have been suggested to exert protective effects against skin aging; however, clinical evidence remains limited.

**Aims:**

This pilot study evaluated the effects of casein hydrolysate MMP1C and whey protein hydrolysate MMP2W on skin condition in healthy Japanese women.

**Methods:**

Two randomized, double‐blind, placebo‐controlled, parallel‐group trials were conducted in women aged 35–54 years who were concerned about facial dullness. In Trial 1, 60 participants were randomly assigned to receive 5 g/day of MMP1C, MMP2W, or placebo for 8 weeks. Skin parameters, including skin color, subjective skin qualities, and advanced glycation end products (AGEs), were assessed. Based on the preliminary findings from Trial 1, skin AGEs were defined as the primary outcome in Trial 2, in which 120 participants received 1 g/day of MMP1C or placebo for 8 weeks. Additional outcomes included ultraviolet (UV) and brown spots.

**Results:**

In Trial 1, 58 participants were included in the efficacy analysis set. Significant improvements in subjective skin qualities were observed in the MMP1C group compared with the placebo group, whereas no significant effects were observed in the MMP2W group. In Trial 2, 98 participants were included in the efficacy analysis set. The MMP1C group showed a significant reduction in AGEs (*p* = 0.014) and suppression of the worsening of UV and brown spots compared with the placebo group. No test food‐related adverse events were reported in either trial.

**Conclusions:**

These findings suggest that MMP1C is a safe functional food ingredient and may contribute to the prevention of skin aging.

## 1 Introduction

The health of the skin, the largest organ of the human body, is closely related to overall well‐being and reflects general health status, vitality, illness, and nutritional condition [1]. Moreover, the visible condition of the skin is a significant health issue, as it affects both physical and mental well‐being. Facial skin tone influences the perception of health [2]; therefore, dullness and spots associated with changes in skin color are common concerns among women. Dullness is related to the degree of coloration caused by melanin deposition, blood flow and circulation, skin texture, and light transmittance [3], while spots reflect uneven pigmentation.

Skin aging, including dullness, is caused by a combination of intrinsic factors such as age and genetics, and extrinsic factors such as ultraviolet (UV) exposure, smoking, and lifestyle habits [4, 5, 6]. For example, glycation stress, caused by the accumulation of advanced glycation end products (AGEs), is one of the main mechanisms underlying skin aging. AGEs are molecules formed via non‐enzymatic reactions between reducing sugars and proteins, lipids, or nucleic acids. Hyperglycemic states and UV exposure accelerate the accumulation of AGEs [7]. Furthermore, several studies have reported a decrease in the density and diameter of blood vessels in the skin due to aging and UV exposure, suggesting a potential association between vascular changes and skin aging [8, 9]. In addition, sleep deprivation has been associated with yellowing of facial skin, and a relationship between sleep quality and skin condition has been reported [10]. Other factors such as oxidative stress and chronic inflammation are also associated with skin aging [11]. Therefore, suppression of these factors is important for the prevention of skin aging.

Oral supplementation has gained attention as an easy method for suppressing skin aging. Various food‐derived ingredients with skin‐improving effects, such as plant extracts and collagen peptides, have been reported [12, 13]. For example, a 12‐week intake of rosemary extract reduced AGEs and alleviated glycation stress, improving skin dullness, texture, erythema, pore size, and overall skin quality [14, 15]. Milk protein‐derived peptides, which have been reported to exhibit various bioactivities, are expected to suppress skin aging. In vivo studies using hairless mice have shown that whey peptide intake reduces melanin levels and suppresses photoaging [16]. In clinical trials, long‐term intake of casein hydrolysate for over 48 weeks has been reported to reduce AGEs and inhibit pigmentation [17]. However, to our knowledge, evidence regarding the effects of milk protein hydrolysates on human skin remains limited.

In this study, we aimed to investigate the effects of an 8‐week intake of casein hydrolysate MMP1C or whey protein hydrolysate MMP2W on skin condition in healthy women with perceived facial skin dullness. In Trial 1, we conducted a preliminary evaluation of each hydrolysate by administering 5 g/day and assessing multiple skin‐related outcomes, including parameters associated with skin tone, as well as AGE levels, skin blood flow, and sleep status. Based on these findings, Trial 2 was subsequently conducted as a hypothesis‐generating exploratory study with a primary focus on skin AGEs. In Trial 2, we evaluated the effects of 1 g/day MMP1C intake.

## 2 Materials and Methods

### 2.1 Trial Design

To investigate the impact of an 8‐week continuous intake of milk protein hydrolysate on the skin, two randomized, placebo‐controlled, double‐blind, parallel‐group trials were conducted at Ueno‐Asagao Clinic (Tokyo, Japan). Trial 1, in which participants consumed 5 g/day of MMP1C, MMP2W, or placebo powder, was conducted between September 2023 and March 2024. Trial 2, in which participants consumed 1 g/day of MMP1C or placebo powder, was conducted between September 2024 and March 2025. These trial protocols were examined and approved by Ueno‐Asagao Clinic Ethical Review Committee on August 31, 2023 (approval code: 2023‐23) and August 26, 2024 (approval code: 2024‐15), respectively, and were conducted in accordance with the Declaration of Helsinki (Fortaleza, revised in 2013) and the Ethical Guidelines for Life Sciences and Medical Research Involving Human Subjects (Ministry of Education, Culture, Sports, Science and Technology, Ministry of Health, Labour and Welfare, and Ministry of Economy, Trade and Industry Notification No. 1, implemented in 2021 and revised in 2023). Prior to the implementation of each trial, the participants were fully informed and confirmed that they fully understood the content of the trial. Consent was obtained from all individuals who wished to participate in the trial. The two trials were registered in the UMIN Clinical Trial Registry (UMIN000052306 and UMIN000055563). No changes were observed in the protocol after the start of the trial.

### 2.2 Participants

In Trial 1, 60 healthy females were selected from those who submitted consent documents. The inclusion criteria were as follows: (1) healthy Japanese females aged 35–54 years, (2) individuals who were healthy and had no chronic physical disease including skin disease; and (3) individuals who were aware of the dullness of their skin. Exclusion criteria were as follows: (1) individuals who had a past or present history of serious illness, (2) individuals who had wounds or inflammation on the evaluation site, (3) individuals with a history of drug or food allergy, (4) individuals who had a habit of continuously ingesting drugs, quasi‐drugs, health functional foods, health foods, or supplements that claim whitening/skin improvement effects within 3 months before the start of the trial, or who planned to use them during the trial period, (5) individuals who had used cosmetics that claim whitening effects within 3 months before the start of the trial, or who planned to use them during the trial period, (6) individuals who were possibly pregnant or lactating, (7) individuals who consumed alcohol excessively (over 60 g/day), (8) individuals who smoked, (9) individuals with possible lifestyle changes during the trial period, (10) individuals who developed seasonal allergy symptoms such as hay fever and suffered from worsening eyes and nose symptoms or used allergy relief drugs during the trial period, (11) individuals who will have gotten sunburned during the trial period, (12) individuals who had undergone a surgical operation or beauty treatment on the evaluation area within 6 months before the start of the trial, (13) individuals who participated in other clinical trials in the past 3 months, and (14) individuals judged to be inappropriate for the trial by the principal investigator. Participants were randomly assigned in a 1:1:1 ratio to the MMP1C, MMP2W, or placebo groups.

In Trial 2, 120 healthy females were selected in the same manner as in Trial 1. The participants were randomly assigned in a 1:1 ratio to the MMP1C or placebo group.

### 2.3 Intervention

The two hydrolysates, MMP1C and MMP2W, were manufactured by Morinaga Milk Industry Co. Ltd. (Tokyo, Japan) through enzymatic hydrolysis of casein and whey protein, respectively. The average molecular weights determined by gel permeation chromatography were 312 Da for MMP1C and 383 Da for MMP2W. The degradation rates, determined using the formol titration method, were 26.8% for MMP1C and 26.4% for MMP2W.

The powdered active food for Trial 1 contained 5 g of MMP1C or MMP2W, whereas the powdered active food for Trial 2 contained 1 g of MMP1C. In both trials, the placebo food contained dextrin instead of hydrolysate. The active and placebo foods were adjusted in flavor by the addition of acidulants, flavorings, and sweeteners to minimize taste differences, and were packaged identically. The participants consumed one sachet dissolved in 100–150 mL of water over an 8‐week period. The participants were encouraged to take the test foods on an empty stomach, that is, immediately after waking up or before meals. Furthermore, the participants were instructed not to alter their lifestyle habits or cosmetics during the trial period and were asked to refrain from consuming health foods that could potentially affect skin quality.

### 2.4 Randomization and Blinding

In Trial 1, the participants were randomly assigned to three groups using a stratified block randomization method with age, L* (indicating skin lightness), and skin color a* (degree of redness) as stratification factors based on the screening test data. Subsequently, it was confirmed that there was no significant divergence among the allocation groups with respect to other indicators. Blinding was implemented after the allocation manager independently confirmed that the three test foods were mutually indistinguishable by coding the test foods using indistinguishable symbols. The prepared allocation list was sealed in an envelope and kept under the strict custody of the allocation manager until all analysis data, methods, and participants were determined. Allocation was blinded to both participants and investigators involved in data collection and analysis.

In Trial 2, the participants were randomly assigned to two groups using a stratified block randomization method with age, AGEs, and skin color L* as stratification factors based on the screening test data. Thereafter, blinding was performed using the same method as that used in Trial 1.

### 2.5 Efficacy Assessment

In Trial 1, the primary outcome was skin color, including L*, a*, b* (degree of yellowness), melanin index, hemoglobin (Hb) index, and Hb oxygen saturation (SO2) index. The secondary outcomes were VISIA skin analysis, visual analog scale (VAS) for skin and other conditions, clinical assessment by dermatologists, skin blood flow, AGE score, and the Japanese version of the Pittsburgh Sleep Quality Index (PSQI‐J).

In Trial 2, the primary outcome was AGE score. Secondary outcomes included the VISIA skin analysis, VAS for skin conditions, clinical assessment by dermatologists, and PSQI‐J.

### 2.6 Measurements

Measurements in both trials were performed at baseline and at weeks 4 and 8 of the intake period. Before the measurements, facial makeup and body cream on the forearms were washed off with cleansers and face washes.

Body weight, body fat percentage, body mass index (BMI), blood pressure, and pulse rate were measured at each time point. Height was measured only at baseline.

The skin color of the cheek was measured using a spectrophotometer (CM‐700d; KONICA MINOLTA JAPAN Inc., Tokyo, Japan) and analysis software (CM‐SA; KONICA MINOLTA JAPAN Inc.).

VISIA analysis of cheek skin was performed using the VISIA Evolution (Canfield Scientific Inc., Parsippany, NJ, USA). Spots, wrinkles, pores, UV spots, brown spots, red areas, and porphyrins were assessed based on the scores calculated from the acquired facial images using dedicated software. A lower score indicated a better skin condition.

The AGE score in the forearm skin, used as a glycation stress marker, was measured with the AGE Reader mu (Diagnoptics Technologies B.V., Groningen, The Netherlands), which is a validated non‐invasive device [18]. The score was determined based on the skin autofluorescence measured by the device.

Skin blood flow in the nailfold capillaries was measured using a TOKU Capillaro (Toku Corp., Tokyo, Japan). The number of blood vessels, blood flow velocity, and vessel diameters (arterial, central, and venous segments) were determined.

The VAS consisted of questions regarding skin condition (skin dullness, spots, texture, elasticity, gloss, clarity, redness, brightness, moisture, and makeup adhesion) and other conditions (facial swelling, fatigue, cold hypersensitivity in the hands and feet, and hair loss). However, in Trial 2, only skin condition was assessed. Participants were asked to rate their most recent condition on a scale of 0 to 100, with 0 representing the best condition and 100 representing the worst condition that they had ever experienced.

Clinical assessments by dermatologists included evaluation of skin texture (skin ridges, skin grooves, and comprehensive evaluation) and skin quality (skin dryness, erythema, scaling, irritation, and itching). Skin texture was rated on a five‐point scale: −2 (poor), −1 (slightly poor), 0 (normal), 1 (slightly good), and 2 (good). Skin quality was evaluated using a five‐point scale as follows: 0 (none; no symptoms), 1 (minimal; barely noticeable symptoms), 2 (mild; slight symptoms), 3 (moderate; obvious symptoms), and 4 (severe; marked symptoms).

The PSQI‐J is a Japanese‐language questionnaire that subjectively assesses sleep quality over the previous month. It generates scores for the following seven components based on the responses: sleep quality, sleep latency, sleep duration, habitual sleep efficiency, sleep disturbances, use of sleeping medication, and daytime dysfunction [19, 20, 21]. Each component was rated on a scale of 0–3 based on the participants' responses, with higher scores indicating a poorer sleep status. The total score, referred to as the PSQI global score (PSQIG), was calculated as the sum of the component scores.

### 2.7 Diary Survey and Compliance Assessment

The participants in each trial recorded their daily intake of the test food, changes in their skin condition and physical health, lifestyle changes, use of medications or dietary supplements, menstrual status, and time spent on outdoor activities (duration of sun exposure) during the trial period. Based on these diary records, study staff conducted interviews before and during the trials to ensure compliance with the protocol requirements.

In Trial 1, noncompliance was predefined as an intake rate of less than 80%, violation of concomitant use restrictions, or any other clearly justified reason for exclusion from analysis. In Trial 2, noncompliance was predefined as an intake rate of less than 80%, significant changes in outdoor activity time, skin‐related problems identified by a physician, or any other clearly justified reason for exclusion from analysis. Changes in outdoor activity time were used to define lifestyle changes. Specifically, the average daily daytime outdoor exposure during the trial was categorized into three groups (0–3 h, 3–6 h, and > 6 h), and participants whose category differed from baseline were considered to have lifestyle changes. In addition, marked fluctuations in outdoor activity during the trial period—defined as a weekly coefficient of variation outside the normal range—were also classified as lifestyle changes. Furthermore, participants with skin‐related problems identified by a physician were considered to have inappropriate measurement conditions, as such conditions may affect the accurate assessment of AGEs.

### 2.8 Safety Assessment

In each trial, all the participants were monitored for adverse events and side effects using daily records and physician interviews during the trial period. Adverse events were graded according to the Common Terminology Criteria for Adverse Events version 5.0, Japanese translation by the Japan Clinical Oncology Group. In addition, the causal relationship between the adverse events and the test food was assessed by a physician.

### 2.9 Sample Size

For Trial 1, the number of participants per group was set at 20, referring to similar clinical trials that examined the effects of food ingredients on the skin [22, 23, 24].

The required sample size for Trial 2 was estimated using G*Power 3.1.9.7 (Heinrich Heine Universität, Düsseldorf, Germany) [25]. Based on the results of AGEs from Trial 1, the effect size was calculated. With a significance level of 5% and statistical power of 90% for comparison between the MMP1C and placebo groups, the required total sample size was determined to be 105 participants. Assuming dropout and withdrawal rates of 12.5%, the number of participants was set at 60 per group.

### 2.10 Statistical Analysis

The safety analysis set included all participants who consumed the test food at least once during the trial period. For efficacy evaluation, Trial 1 included all participants who provided informed consent, met the eligibility criteria, and had available randomization data. Trial 2 was designed to assess the effect of the intervention under adequate adherence; therefore, the efficacy analysis was conducted in the per‐protocol set, excluding participants who met prespecified criteria for noncompliance.

To analyze participant backgrounds, we performed the Student's *t*‐test for quantitative data, Wilcoxon rank‐sum test for ordinal data, and Fisher's exact test for categorical data. To compare the primary and secondary outcomes between the groups, analysis of covariance was performed, with the 8‐week value as the response variable, the test food group as the explanatory variable, and the baseline value as the covariate. Fisher's protected least significant difference post hoc test was used to adjust for multiplicity among the three groups in Trial 1. A closed‐testing procedure was used to multiply the measurement time points in the tests. Specifically, if a significant difference was observed at 8 weeks, the same test was conducted at 4 weeks. Comparisons between baseline and each time point within the groups were performed using a paired *t*‐test. Intergroup comparisons of changes at each time point during the intake period were conducted using the Student's *t*‐test as an exploratory analysis. Test food intake rates were compared between the groups using the Wilcoxon rank‐sum test. The number and incidence of adverse events and side effects in each group during the trial period were counted. The incidence of adverse events was compared between the active food and placebo groups using Fisher's exact test. All tests were two‐tailed, and the statistical significance level was set at *p* < 0.05.

Data were tabulated using Microsoft Office Excel 2016 (Microsoft Corp., Redmond, WA, USA) and descriptive statistics were calculated and analyzed using SPSS version 26 (IBM Corp., New York, USA) and SAS 9.4 (SAS Institute Inc., Cary, NC, USA).

## 3 Results

### 3.1 Participants in Trial 1

**FIGURE 1:**
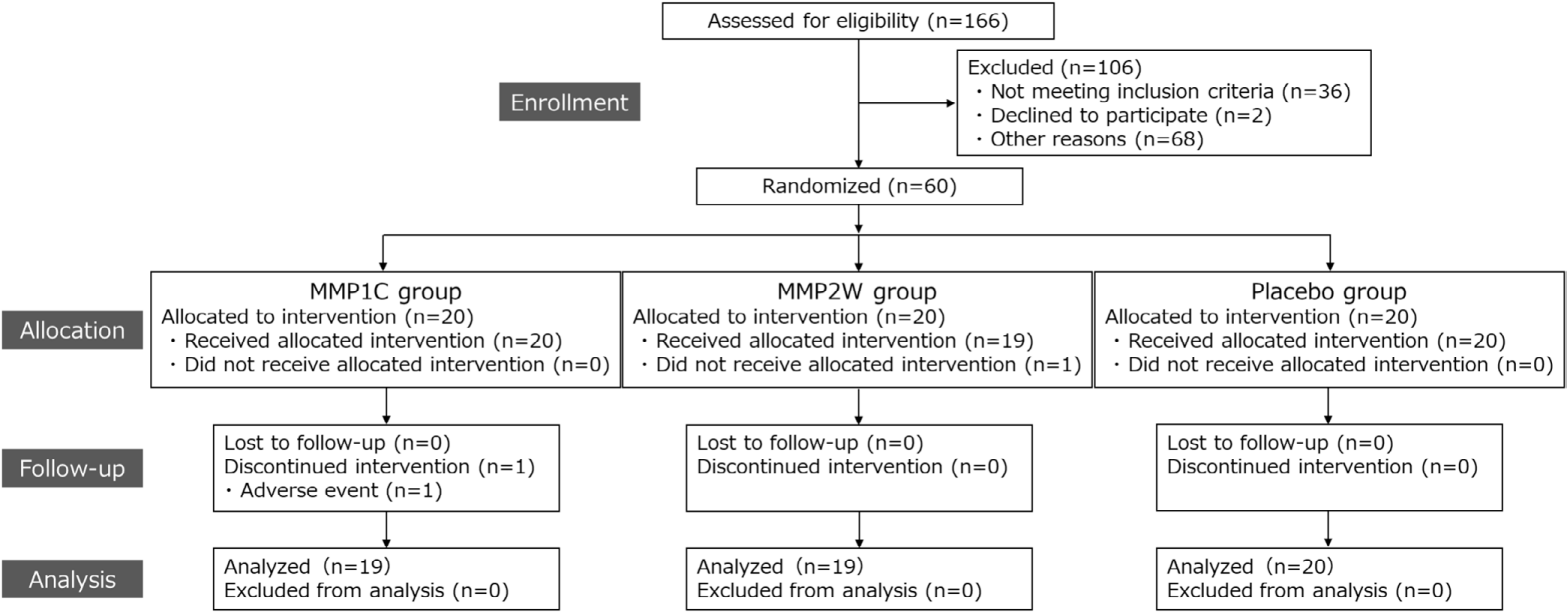
CONSORT flow diagram of Trial 1.

Of the 166 participants assessed for eligibility, 60 were enrolled and randomly allocated to one of the three groups: 20 participants to the MMP1C group, 20 participants to the MMP2W group, and 20 participants to the placebo group, respectively (Figure 1). The population for safety analysis included 59 participants, excluding one participant who dropped out for personal reasons unrelated to the trial before the intervention. After the intervention, one participant dropped out because of an adverse event unrelated to the test food, resulting in 19 participants in the MMP1C group, 19 in the MMP2W group, and 20 in the placebo group completing the trial. The population for efficacy analysis included 58 participants. There were no significant differences in the intake rate of the test food between each hydrolysate group and the placebo group (MMP1C group; 99.2% ± 2.6%, MMP2W group; 99.3% ± 2.9%, Placebo group; 99.8% ± 0.6%, *p* > 0.05). The baseline characteristics of the participants are presented in Table 1. There were no significant differences between the hydrolysate and placebo groups in terms of age, body measurements (BMI, blood pressure, and pulse rate), or skin color (L*, a*, b*, melanin index, Hb index, and Hb SO2 index).

**TABLE 1:** Participant characteristics in Trial 1.

Characteristic	MMP1C (*n* = 19)	MMP2W (*n* = 19)	Placebo (*n* = 20)
Age (years)	45.3 ± 5.7	45.4 ± 5.4	45.5 ± 4.0
BMI (kg/m^2^)	20.9 ± 2.5	20.6 ± 2.7	20.3 ± 2.8
Systolic blood pressure (mmHg)	102.7 ± 10.6	103.9 ± 7.0	103.7 ± 10.3
Diastolic blood pressure (mmHg)	67.4 ± 8.3	65.3 ± 7.4	63.4 ± 9.1
Pulse rate (bpm)	74.4 ± 11.0	70.3 ± 8.9	72.6 ± 11.2
L*	65.96 ± 2.00	66.03 ± 2.33	66.30 ± 2.86
a*	8.78 ± 1.11	8.43 ± 1.31	8.64 ± 1.17
b*	18.57 ± 1.44	18.49 ± 1.61	18.40 ± 1.36
Melanin index	1.13 ± 0.12	1.10 ± 0.15	1.11 ± 0.15
Hb index	1.03 ± 0.26	1.02 ± 0.22	1.00 ± 0.27
Hb SO2 index (%)	55.68 ± 7.63	54.10 ± 6.24	56.53 ± 7.58
AGE score (AU)	1.99 ± 0.30	2.03 ± 0.32	2.01 ± 0.34

*Note:* Data represent mean ± standard deviation.

Abbreviations: a*, degree of redness; AGE, advanced glycation end products; AU, arbitrary unit; b*, degree of yellowness; BMI, body mass index; bpm, beats per minute; Hb, hemoglobin; L*, skin lightness; SO2, oxygen saturation.

At week 4, data for one participant in the MMP1C group were missing because measurements other than the VAS and PSQI‐J were not performed.

### 3.2 Outcomes in Trial 1

Skin color data at baseline, week 4, and week 8 are shown in Table 2. After 8 weeks, no significant improvements were observed in any parameter of this item in the MMP1C or MMP2W groups compared with the placebo group. The a*, b*, melanin index, and Hb index showed similar changes at week 8 compared with the baseline in all groups. However, at week 8, the Hb SO2 index significantly only increased in the MMP2W group compared with the baseline.

**TABLE 2:** Assessment of skin color by the spectrophotometer in Trial 1.

	Group	Baseline	Week 4	Week 8
L*	MMP1C	65.96 ± 2.00	65.90 ± 2.40	65.69 ± 2.19
	MMP2W	66.03 ± 2.33	66.21 ± 2.47	65.88 ± 2.46
	Placebo	66.30 ± 2.86	66.79 ± 2.72*	66.54 ± 2.89
a*	MMP1C	8.78 ± 1.11	9.06 ± 1.43	9.43 ± 1.42**
	MMP2W Placebo	8.43 ± 1.31 8.64 ± 1.17	8.79 ± 1.36** 8.76 ± 1.18	9.25 ± 1.56*** 9.07 ± 1.44*
b*	MMP1C	18.57 ± 1.44	18.05 ± 1.51	17.41 ± 1.63***
	MMP2W	18.49 ± 1.61	17.99 ± 1.59**	17.42 ± 1.79***
	Placebo	18.40 ± 1.36	17.83 ± 1.35**	17.25 ± 1.26***
Melanin index	MMP1C	1.13 ± 0.12	1.09 ± 0.14*	1.07 ± 0.15***
	MMP2W	1.10 ± 0.15	1.07 ± 0.16**	1.05 ± 0.16**
	Placebo	1.11 ± 0.15	1.06 ± 0.14**	1.04 ± 0.13***
Hb index	MMP1C	1.03 ± 0.26	1.13 ± 0.27*	1.25 ± 0.26***
	MMP2W	1.02 ± 0.22	1.11 ± 0.22***	1.22 ± 0.25***
	Placebo	1.00 ± 0.27	1.07 ± 0.24*	1.17 ± 0.26***
Hb SO2 index (%)	MMP1C	55.68 ± 7.63	57.31 ± 7.11	56.27 ± 5.89
	MMP2W	54.10 ± 6.24	56.41 ± 5.74**	58.03 ± 6.22***
	Placebo	56.53 ± 7.58	58.43 ± 5.73	58.44 ± 5.24

*Note:* Data represent mean ± standard deviation. The sample sizes were as follows: MMP1C group, *n* = 19 (week 4, *n* = 18); MMP2W group, *n* = 19; and placebo group, *n* = 20.

Abbreviations: a*, degree of redness; ANCOVA, analysis of covariance; b*, degree of yellowness; Hb, hemoglobin; L*, skin lightness; SO2, oxygen saturation.

* *p* < 0.05.

** *p* < 0.01.

*** *p* < 0.001 compared with baseline using the paired *t*‐test. There were no significant differences between the groups by ANCOVA adjusted for baseline, with Fisher's protected least significant difference post hoc test and the closed‐testing procedure.

The skin quality data assessed using the VAS at baseline, week 4, and week 8 are presented in Table 3. After 8 weeks, the MMP1C group showed significant improvements in six parameters of this item compared with the placebo group: skin dullness (*p* = 0.010), skin spots (*p* = 0.021), skin texture (*p* = 0.005), skin elasticity (*p* = 0.005), skin gloss (*p* = 0.003), and skin clarity (*p* = 0.012). After 4 weeks, there was no significant difference in any parameters between the MMP1C and placebo groups. All parameters, except skin redness, improved at weeks 4 and 8 compared with the baseline in all three groups. Furthermore, no significant differences between the groups were observed for any parameter other than skin quality, as assessed by the VAS (Table S1).

**TABLE 3:** Assessment of skin quality by VAS in Trial 1.

	Group	Baseline	Week 4	Week 8
Skin dullness	MMP1C	67.2 ± 17.2	49.5 ± 12.6**	35.1 ± 13.6*** †
	MMP2W	69.8 ± 14.2	52.9 ± 17.4**	50.5 ± 18.5**
	Placebo	74.8 ± 11.6	52.8 ± 9.3***	48.8 ± 12.4***
Skin spots	MMP1C	67.9 ± 15.0	50.4 ± 13.4**	40.2 ± 12.0*** †
	MMP2W	74.4 ± 11.0	55.3 ± 17.8***	55.2 ± 19.4***
	Placebo	74.2 ± 11.2	55.5 ± 11.6***	53.9 ± 13.7***
Skin texture	MMP1C	69.3 ± 14.8	47.9 ± 16.1***	37.5 ± 12.5*** ††
	MMP2W	67.8 ± 16.1	49.2 ± 14.2**	49.5 ± 19.5**
	Placebo	71.8 ± 11.3	53.1 ± 10.9***	51.4 ± 10.6***
Skin elasticity	MMP1C	70.2 ± 14.3	48.7 ± 16.1***	36.7 ± 12.7*** ††
	MMP2W	71.1 ± 17.1	50.9 ± 16.9**	50.7 ± 21.0**
	Placebo	73.5 ± 14.4	52.3 ± 10.9***	52.2 ± 13.6***
Skin gloss	MMP1C	66.8 ± 12.1	45.8 ± 13.2***	34.6 ± 14.5*** ††
	MMP2W	73.5 ± 11.2	49.9 ± 13.5***	50.2 ± 21.9***
	Placebo	71.1 ± 17.4	52.2 ± 12.5**	51.7 ± 13.4***
Skin clarity	MMP1C	74.2 ± 10.8	48.9 ± 13.9***	36.1 ± 15.7*** †
	MMP2W	78.0 ± 8.6	53.2 ± 16.3***	54.8 ± 20.4***
	Placebo	76.1 ± 9.3	53.1 ± 13.3***	51.0 ± 15.4***
Skin redness	MMP1C	49.1 ± 19.8	42.6 ± 12.0	39.0 ± 14.0
	MMP2W	53.0 ± 15.8	44.6 ± 14.7	44.1 ± 17.1
	Placebo	43.9 ± 20.2	44.1 ± 15.6	40.2 ± 16.4
Skin	MMP1C	66.5 ± 11.0	44.9 ± 10.6***	37.1 ± 14.5***
brightness	MMP2W	70.8 ± 10.6	49.7 ± 16.0***	49.9 ± 18.4***
	Placebo	70.8 ± 11.0	51.1 ± 11.1***	45.9 ± 12.5***
Skin moisture	MMP1C	68.5 ± 12.2	42.8 ± 15.3***	38.1 ± 18.0***
	MMP2W	67.6 ± 12.6	47.1 ± 17.6***	53.2 ± 20.2*
	Placebo	67.3 ± 15.3	51.1 ± 18.7**	47.3 ± 13.6***
Makeup adhesion	MMP1C	66.4 ± 12.7	44.6 ± 11.5***	37.0 ± 15.7***
	MMP2W	66.6 ± 13.4	49.2 ± 17.4**	53.1 ± 19.5*
	Placebo	67.9 ± 10.3	52.2 ± 16.5***	47.4 ± 13.8***

*Note:* Data represent mean ± standard deviation. The sample sizes were as follows: MMP1C group, *n* = 19; MMP2W group, *n* = 19; and placebo group, *n* = 20.

Abbreviations: ANCOVA, analysis of covariance; VAS, visual analog scale.

* *p* < 0.05.

** *p* < 0.01.

*** *p* < 0.001 compared with baseline using the paired *t*‐test.

^†^ *p* < 0.05.

^††^ *p* < 0.01 compared with the placebo group by ANCOVA adjusted for baseline, with Fisher's protected least significant difference post hoc test and the closed‐testing procedure.

In the assessment of skin quality by VISIA and dermatologists, AGEs, skin blood flow, and PSQI‐J, no significant differences were found between the hydrolysate and placebo groups at any time point (Tables S2–S5). However, in the PSQI‐J evaluation, the MMP1C group showed improvements in subjective sleep quality and sleep duration from baseline to week 8, with improvements in sleep latency and PSQI‐G observed at weeks 4 and 8. Regarding the AGE score, in the results of the *t*‐test of least squares means between the MMP1C and placebo groups at week 8, a tendency toward decreased AGEs was observed in the MMP1C group (*p* = 0.084). Furthermore, the AGE score in the MMP1C group tended to decrease at week 4 (1.92 ± 0.33, *p* = 0.081) and week 8 (1.93 ± 0.29, *p* = 0.116) compared with the baseline (1.99 ± 0.30). The between‐group difference did not reach statistical significance; however, the effect size was small‐to‐moderate (partial *η* ^*2*^ = 0.093; standardized mean difference based on adjusted means = −0.27). Based on the findings of Trial 1, Trial 2 was subsequently conducted as a hypothesis‐generating exploratory study with a particular emphasis on skin AGEs.

### 3.3 Safety in Trial 1

During the 8‐week intake period, 72 adverse events were reported among the 34 participants. Specifically, the MMP1C group accounted for 28 events in 15 participants, the MMP2W group for 22 events in 11 participants, and the placebo group for 22 events in 8 participants. Physicians assessed these events and determined that all were transient symptoms or were attributable to external factors, with mild and non‐severe symptoms. Therefore, it was concluded that there were no side effects associated with the test food. No significant differences were observed in the incidence of adverse events between the hydrolysate and placebo groups (MMP1C group; 75.0%; MMP2W group; 57.9%; Placebo group; 40.0%; *p* > 0.05).

### 3.4 Participants in Trial 2

**FIGURE 2:**
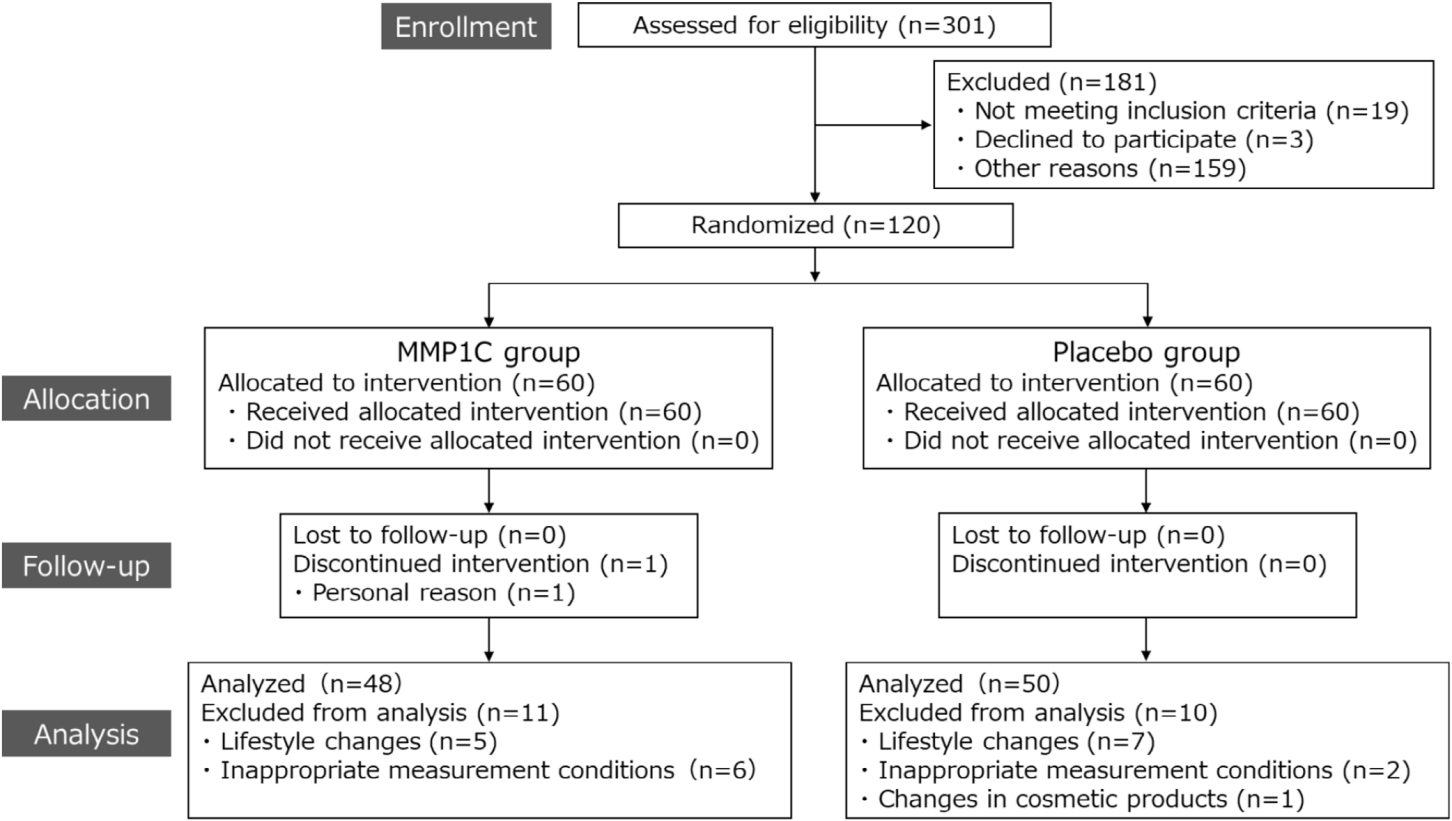
CONSORT flow diagram of Trial 2.

Of the 301 participants assessed for eligibility, 120 were enrolled and randomly allocated to one of two groups: 60 participants to the MMP1C group and 60 participants to the placebo group (Figure 2). The population for safety analysis included 120 participants. After the intervention, one participant dropped out for personal reasons, resulting in 59 participants in the MMP1C group and 60 participants in the placebo group completing the trial. The analysis population for the MMP1C group consisted of 48 participants, after excluding 11 participants who exhibited lifestyle changes or inappropriate measurement conditions during the trial period. For the placebo group, the analysis population consisted of 50 participants, excluding 10 participants who exhibited lifestyle changes, inappropriate measurement conditions, or changes in cosmetic products during the trial period.

There were no significant differences in the intake rate of the test food between each hydrolysate group and the placebo group (MMP1C group; 99.9% ± 0.7%, Placebo group; 99.9% ± 0.9%, *p* > 0.05). The baseline characteristics of the participants are presented in Table 4. There were no significant differences between the MMP1C and placebo groups in terms of age, body measurements (BMI, blood pressure, and pulse rate), or AGE scores.

**TABLE 4:** Participant characteristics in Trial 2.

Characteristic	MMP1C (*n* = 48)	Placebo (*n* = 50)
Age (years)	46.3 ± 5.8	46.9 ± 4.7
BMI (kg/m^2^)	20.5 ± 3.3	20.6 ± 2.7
Systolic blood pressure (mmHg)	107.9 ± 10.0	107.4 ± 11.3
Diastolic blood pressure (mmHg)	67.0 ± 9.6	67.0 ± 8.2
Pulse rate (bpm)	69.6 ± 9.2	67.3 ± 8.6
AGE score (AU)	2.01 ± 0.27	2.02 ± 0.26

*Note:* Data represent mean ± standard deviation.

Abbreviations: AGE, advanced glycation end products; AU, arbitrary unit; BMI, body mass index.

At week 4, data were missing for one participant in the placebo group because no measurements were performed.

### 3.5 Outcomes in Trial 2

**FIGURE 3:**
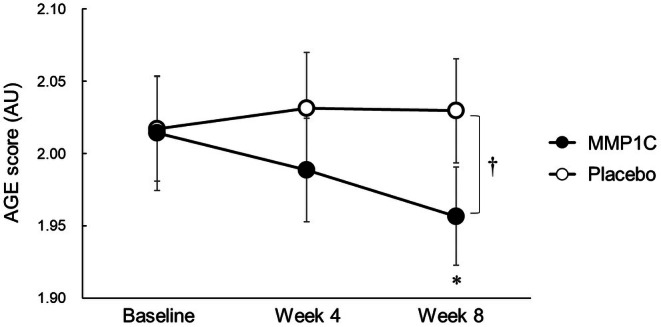
AGE score of each group during the treatment period in Trial 2. The data at each specific time point represent mean ± standard error of the mean. The sample sizes were as follows: MMP1C group, *n* = 48; and placebo group, *n* = 50 (week 4, *n* = 49). *p < 0.05 compared with baseline using the paired *t*‐test. ^†^ *p* < 0.05 compared with the placebo group by ANCOVA adjusted for baseline and the closed‐testing procedure. AGE, advanced glycation end products; ANCOVA, analysis of covariance; AU, arbitrary unit.

Figure 3 shows the AGEs at baseline, week 4, and week 8. In the MMP1C group, the scores were 2.01 ± 0.28 at baseline, 1.99 ± 0.25 at week 4, and 1.96 ± 0.24 at week 8. In the placebo group, the scores were 2.02 ± 0.26 at baseline, 2.03 ± 0.27 at week 4, and 2.03 ± 0.25 at week 8. After 8 weeks, the AGE score in the MMP1C group was significantly lower than that in the placebo group (*p* = 0.014, partial *η* ^*2*^ = 0.061, standardized mean difference based on adjusted means = −0.29). No significant differences were observed between the groups at week 4. In addition, after 8 weeks, AGEs in the MMP1C group significantly decreased compared with the baseline (*p* = 0.004), whereas no significant change was observed in the placebo group.

**FIGURE 4:**
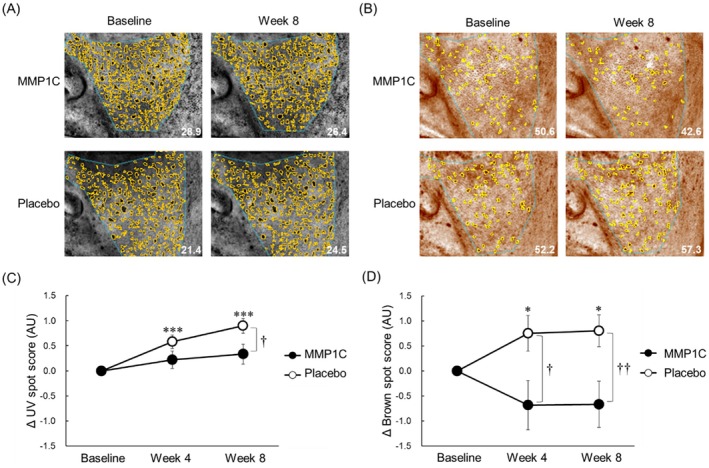
Effects of MMP1C on UV spots and brown spots in Trial 2. Representative VISIA images of the left cheek from participants: (A) UV spot scoring images and (B) brown spot scoring images obtained at baseline and at week 8 after intake. The score values are shown at the bottom of each image. (C) Changes in UV spot score and (D) brown spot score over the 8‐week period. The data represent mean ± standard error of the mean. The sample sizes were as follows: MMP1C group, *n* = 48; and placebo group, *n* = 50 (week 4, *n* = 49). *p < 0.05 and ***p < 0.001 compared with baseline using the paired *t*‐test. ^†^ *p* < 0.05 and ^††^ *p* < 0.01 compared with the placebo group using the Student's *t*‐test. AU, arbitrary unit; UV, ultraviolet.

Table 5 shows the skin quality data assessed using the VISIA at baseline, week 4, and week 8. In the MMP1C group, except for a significant improvement in the red areas (*p* = 0.013), no significant differences were observed at week 4 compared with the baseline. In contrast, in the placebo group, significant increases in the scores for spots at week 8 (*p* = 0.040), texture at week 4 (*p* = 0.009), pores at weeks 4 and 8 (*p* < 0.001 and *p* = 0.016, respectively), UV spots at weeks 4 and 8 (*p* < 0.001 and *p* < 0.001, respectively), and brown spots at weeks 4 and 8 (*p* = 0.040 and *p* = 0.014, respectively) were observed compared with the baseline, indicating deterioration. Furthermore, the MMP1C group showed significant improvements in UV spots at week 8 (*p* = 0.019) and brown spots at weeks 4 and 8 (*p* = 0.022 and *p* = 0.011, respectively) compared with the placebo group. Similar results were confirmed in the representative cheek images of UV‐induced spots and brown spots (Figure 4A,B). The changes observed at each time point also clearly indicated that, while these two types of spots increased in the placebo group, no increase was observed in the MMP1C group (Figure 4C,D).

**TABLE 5:** Assessment of skin quality by VISIA in Trial 2.

	Group	Baseline	Week 4	Week 8
Spots	MMP1C	31.9 ± 10.4	32.4 ± 10.2	31.6 ± 11.0
	Placebo	31.7 ± 8.3	32.3 ± 8.3	33.2 ± 8.7*
Wrinkles	MMP1C	35.7 ± 30.5	35.1 ± 28.8	32.3 ± 28.1
	Placebo	27.5 ± 20.8	31.0 ± 21.8	28.3 ± 20.9
Texture	MMP1C	10.0 ± 6.6	10.5 ± 6.4	10.0 ± 6.3
	Placebo	9.3 ± 4.8	10.3 ± 5.5**	9.5 ± 5.0
Pores	MMP1C	20.6 ± 12.7	22.0 ± 13.4	22.1 ± 13.5
	Placebo	21.4 ± 15.1	23.0 ± 15.9***	23.2 ± 14.9*
UV spots	MMP1C	29.3 ± 5.1	29.5 ± 4.8	29.6 ± 5.1^†^
	Placebo	29.8 ± 4.6	30.4 ± 4.7***	30.7 ± 4.5***
Brown spots	MMP1C	57.3 ± 6.6	56.6 ± 7.8^†^	56.6 ± 6.8^†^
	Placebo	56.7 ± 6.2	57.7 ± 5.9*	57.5 ± 6.2*
Red areas	MMP1C	38.4 ± 8.8	36.5 ± 9.4*	38.0 ± 10.4
	Placebo	37.3 ± 8.2	37.7 ± 10.2	38.6 ± 9.4
Porphyrins	MMP1C	7.6 ± 7.5	6.9 ± 6.7	6.5 ± 7.9
	Placebo	7.5 ± 6.4	6.9 ± 5.3	6.8 ± 5.2

*Note:* Data represent mean ± standard deviation. The sample sizes were as follows: MMP1C group, *n* = 48; and placebo group, *n* = 50 (week 4, *n* = 49).

Abbreviations: ANCOVA, analysis of covariance; UV, ultraviolet.

* *p* < 0.05.

** *p* < 0.01.

*** *p* < 0.001 compared with baseline using the paired *t*‐test.

^†^ *p* < 0.05 compared with the placebo group by ANCOVA adjusted for baseline and the closed‐testing procedure.

In the assessment of skin color by the spectrophotometer and skin quality by dermatologists, no significant differences were found between the MMP1C and placebo groups for any of the parameters (Tables S6 and S7). Skin quality assessed by VAS showed significant improvement at weeks 4 and 8 compared with the baseline in both groups; however, there was no significant difference between the groups (Table S8). Furthermore, no effects on sleep due to MMP1C intake were identified (Table S9).

### 3.6 Safety in Trial 2

During the 8‐week intake period, 29 adverse events were reported among the 27 participants. Specifically, the MMP1C group accounted for 17 events in 16 participants and the placebo group accounted for 12 events in 11 participants. Physicians assessed these events and determined that all were transient symptoms or were attributable to external factors, with mild and non‐severe symptoms. Therefore, it was concluded that there were no side effects associated with the test food. No significant differences were observed in the incidence of adverse events between the MMP1C and placebo groups (MMP1C group; 26.7%; Placebo group; 18.3%; *p* > 0.05).

## 4 Discussion

The present study showed that an 8‐week intake of MMP1C had beneficial effects on skin quality. In Trial 1, daily intake of 5 g of MMP1C was found to improve subjective skin quality, including skin dullness and skin spots. No similar improvements were observed after MMP2W intake, suggesting that these effects were specific to MMP1C. Furthermore, a tendency toward reduced AGEs was observed with MMP1C intake. As AGEs are considered a biologically meaningful indicator of skin aging and Trial 1 demonstrated a consistent downward trend in AGE levels, AGEs were exploratorily defined as the primary outcome for Trial 2. In Trial 2, daily intake of 1 g of MMP1C reduced skin AGEs. In addition, an inhibitory effect on the worsening of UV and brown spots was observed, suggesting that the intervention may help prevent damage associated with photoaging.

The difference in the results of the subjective skin quality assessment using the VAS between Trials 1 and 2 may be due to the difference in intake dosage. In both trials, significant improvements were observed in most parameters, even in the placebo group, indicating a strong placebo effect. While the effects of 5 g/day of MMP1C exceeded those of placebo, the effects of 1 g/day of MMP1C might have been less distinguishable from the placebo effect, possibly owing to the lower dosage being insufficient to produce a measurable difference. To clarify the subjective effects of low‐dose MMP1C, it would be beneficial to provide participants with sufficient training on the VAS and extend the intake period.

The differences in AGEs outcomes between the two trials may be attributable to variations in sample size and sun exposure. Given substantial inter‐individual variability, Trial 2 was conducted with an appropriately powered sample size, calculated based on the results of Trial 1, to evaluate the effects of MMP1C on AGEs. Although Trial 1 did not reach statistical significance, the observed effect size (standardized mean difference) was comparable between the two trials, suggesting consistency in the direction of the effect. The lack of significance in Trial 1 may therefore be partly explained by limited statistical power. In addition, UVA exposure is well known to induce the accumulation of AGEs in the skin [7]. In Trial 1, some participants exhibited changes in the duration of sun exposure between the pre‐trial and the trial period or within the trial period, which may have influenced the assessment of efficacy. Therefore, in Trial 2, participants who showed marked changes in daytime outdoor activity time were excluded from the efficacy analysis set. Based on the above, the results of Trial 2 suggest that MMP1C may be effective in participants with relatively stable daily outdoor exposure. However, as outdoor activity time can vary substantially in real‐life situations, caution should be exercised when generalizing the results of this study. These findings suggest that further studies are required to clarify whether the observed reduction in AGEs is attributable to the intervention itself or to its combination with lifestyle‐related behavioral changes, such as adjustments in outdoor activity time. Similar considerations may also apply to the differences observed in UV and brown spots, given that UVA exposure contributes to pigmentation [7].

In Trial 2, a modest change of 0.05 in the skin AGE score was observed after 8 weeks of MMP1C intake. Previous reports have shown that skin AGE scores measured by skin autofluorescence in non‐smokers increase linearly with age at a rate of 0.023 per year [26]. Based on this estimate, the observed change in the skin AGE score may correspond to the equivalent of 2.2 years of glycation‐related aging. However, caution is warranted in interpreting this finding as evidence of a definitive anti‐aging effect. Future studies incorporating more comprehensive assessments, including blood and skin biopsy measurements of AGEs, are needed to better elucidate the clinical significance of these findings.

MMP1C, by reducing AGEs, may lead to beneficial effects on objective UV spots and brown spots as well as subjective improvements in skin quality. UV spot evaluation utilizes images obtained under UV illumination, exploiting the characteristics of the epidermal melanin just beneath the skin surface to selectively absorb UV light. Brown spot evaluation uses images under orthogonal cross‐polarized light illumination, enabling the detection of deeper melanin deposits that are undetectable in UV images [27]. Thus, both spots reflect melanin deposition. A significant positive correlation was observed between AGEs and melanin content [28]. It has been reported that AGE accumulation promotes melanin production by activating AGE receptors on melanocytes and by activating NLRP3 inflammasomes in fibroblasts, thereby increasing interleukin‐18 production and secretion [29, 30]. Based on these previous findings, we speculate that MMP1C intake suppressed the worsening of UV and brown spots by reducing AGEs. Furthermore, AGEs induce skin stiffening and reduce elasticity by cross‐linking with components of the extracellular matrix, including collagen and elastin [7]. Glycated elastin fibers abnormally aggregate and interact excessively with lysozymes in skin affected by solar elastosis, whereas such phenomena are not observed in sun‐protected areas, suggesting the involvement of both glycation and photoaging [31]. Therefore, it is conceivable that MMP1C improves various subjective qualities of skin by reducing AGEs, indicating its potential as an effective ingredient for the prevention of skin aging.

Although sleep status is associated with AGE accumulation [32], no significant difference was observed in PSQI‐J scores between the MMP1C and placebo groups in either trial. Nevertheless, in Trial 1, the MMP1C group showed improvements in several components, including PSQIG, compared with the baseline. As the baseline PSQIG values were less than 5, analyzing participants with higher baseline values or sleep difficulties may clarify the effects of MMP1C on sleep.

Because all participants in both trials were Japanese females aged 35–54 years, the effects of MMP1C on the skin of other populations remain unclear. Previous studies have reported that major Northeast Asian populations, including Japanese, Korean, and Han Chinese, are genetically closely related [33], suggesting that similar effects to those observed in the present study may also be expected in these populations. Future studies including a broader range of ethnicities, age groups, and males will be valuable for further elucidating the scope and relevance of the effects of MMP1C on skin health.

The major strengths of our study were its well‐controlled trial design and adequate sample size to detect a relatively small but potentially clinically significant change in AGEs. However, these trials have some limitations. First, the exploratory design of the study, which was conducted in a specific participant population and under specific conditions, limits the generalizability of the findings. Second, the short intervention period precluded evaluation of long‐term effects. Third, skin AGEs were assessed using a surrogate marker rather than direct biochemical measurements. Future confirmatory studies are warranted to address these limitations.

In conclusion, this pilot study, comprising two trials, suggests that an 8‐week intake of MMP1C may safely and effectively exert multiple beneficial effects on the skin by reducing AGEs.

## Author Contributions

**Sayuri Arai:** conceptualization, methodology, validation, formal analysis, investigation, data curation, writing – original draft, visualization. **Naoki Yuda:** conceptualization, methodology, validation, investigation, data curation, writing – review and editing, project administration. **Masaki Kurimoto:** conceptualization, methodology, validation, investigation, writing – review and editing. **Ryota Suzuki:** validation, writing – review and editing. **Hajime Nakada:** conceptualization, resources, writing – review and editing, project administration. **Hiroshi Ochi:** conceptualization, resources, writing – review and editing, project administration. **Miyuki Tanaka:** conceptualization, methodology, writing – review and editing, supervision, funding acquisition. **Atsushi Nakajima:** investigation, resources, data curation, writing – review and editing, project administration. **Yuki Yamamoto:** validation, writing – review and editing, supervision. **Masatoshi Jinnin:** validation, writing – review and editing, supervision.

## Funding

This work was supported by Morinaga Milk Industry.

## Ethics Statement

The protocols for the two trials in this study were approved by the Ueno‐Asagao Clinic Ethical Review Committee (Tokyo, Japan) on August 31, 2023 (approval code: 2023–23) and August 26, 2024 (approval code: 2024–15), respectively. Written informed consent was obtained from all participants before their inclusion. The two trials were registered in the UMIN Clinical Trial Registry as UMIN000052306 and UMIN000055563.

## Conflicts of Interest

S.A., N.Y., M.K., R.S., H.N., H.O., and M.T. are employees of Morinaga Milk Industry Co. Ltd. The rest of the authors declare no conflicts of interest.

## Supporting information


**Table S1:** Assessment of facial swelling, fatigue, cold hypersensitivity in the hands and feet, and hair loss by VAS in Trial 1.
**Table S2:** Assessment of skin quality by VISIA in Trial 1.
**Table S3:** Assessment of skin quality by dermatologists in Trial 1.
**Table S4:** Assessment of glycation stress marker and skin blood flow in Trial 1.
**Table S5:** Assessment of sleep quality by PSQI‐J in Trial 1.
**Table S6:** Assessment of skin quality by the spectrophotometer in Trial 2.
**Table S7:** Assessment of skin quality by dermatologists in Trial 2.
**Table S8:** Assessment of skin quality by VAS in Trial 2.
**Table S9:** Assessment of sleep quality by PSQI‐J in trial 2.

## Data Availability

The data that support the findings of this study are available from the corresponding author upon reasonable request.

## References

[1] S. Humphrey , S. Manson Brown , S. J. Cross , and R. Mehta , “Defining Skin Quality: Clinical Relevance, Terminology, and Assessment,” Dermatologic Surgery 47, no. 7 (2021): 974–981, 10.1097/DSS.0000000000003079. 34148998 PMC8231670

[2] I. D. Stephen , M. J. Law Smith , M. R. Stirrat , and D. I. Perrett , “Facial Skin Coloration Affects Perceived Health of Human Faces,” International Journal of Primatology 30, no. 6 (2009): 845–857, 10.1007/s10764-009-9380-z. PMC2780675 19946602

[3] M. Naganuma , “History of the Effectiveness of Cosmetics,” Journal of Japanese Cosmetic Science Society 39, no. 4 (2015): 275–285.

[4] M. Chaudhary , A. Khan , and M. Gupta , “Skin Ageing: Pathophysiology and Current Market Treatment Approaches,” Current Aging Science 13, no. 1 (2020): 22–30, 10.2174/1567205016666190809161115. 31530270 PMC7403684

[5] S. H. Shin , Y. H. Lee , N. K. Rho , and K. Y. Park , “Skin Aging From Mechanisms to Interventions: Focusing on Dermal Aging,” Frontiers in Physiology 14 (2023): 1195272, 10.3389/fphys.2023.1195272. 37234413 PMC10206231

[6] N. Dorf and M. Maciejczyk , “Skin Senescence‐From Basic Research to Clinical Practice,” Frontiers in Medicine 11 (2024): 1484345, 10.3389/fmed.2024.1484345. 39493718 PMC11527680

[7] W. Zheng , H. Li , Y. Go , X. H. F. Chan , Q. Huang , and J. Wu , “Research Advances on the Damage Mechanism of Skin Glycation and Related Inhibitors,” Nutrients 14, no. 21 (2022): 4588, 10.3390/nu14214588. 36364850 PMC9655929

[8] Y. Hara , T. Yamashita , and K. Kikuchi , “Visualization of Age‐Related Vascular Alterations in Facial Skin Using Optical Coherence Tomography‐Based Angiography,” Journal of Dermatological Science 90, no. 1 (2018): 96–98, 10.1016/j.jdermsci.2017.11.018. 29373160

[9] X. Li , T. Matsumoto , and M. Takuwa , “Protective Effects of Astaxanthin Supplementation Against Ultraviolet‐Induced Photoaging in Hairless Mice,” Biomedicine 8, no. 2 (2020): 18, 10.3390/biomedicines8020018. PMC7168265 31973028

[10] A. Matsubara , G. Deng , and L. Gong , “Sleep Deprivation Increases Facial Skin Yellowness,” Journal of Clinical Medicine 12, no. 2 (2023): 615, 10.3390/jcm12020615. 36675544 PMC9861417

[11] F. Papaccio , A. D'Arino , S. Caputo , and B. Bellei , “Focus on the Contribution of Oxidative Stress in Skin Aging,” Antioxidants 11, no. 6 (2022): 1121, 10.3390/antiox11061121. 35740018 PMC9220264

[12] R. Geng , S. G. Kang , K. Huang , and T. Tong , “Boosting the Photoaged Skin: The Potential Role of Dietary Components,” Nutrients 13, no. 5 (2021): 1691, 10.3390/nu13051691. 34065733 PMC8156873

[13] N. Tranchida , F. Molinari , G. A. Franco , M. Cordaro , and R. Di Paola , “Potential Role of Dietary Antioxidants During Skin Aging,” Food Science & Nutrition 13, no. 5 (2025): e70231, 10.1002/fsn3.70231. 40321615 PMC12046069

[14] A. Guiotto , A. Pecorelli , Z. D. Draelos , A. Gueniche , M. Yatskayer , and D. B. Nelson , “Reversing Oxinflammation Associated With Glycative Stress and Formation of Advanced Glycation End Products With a Dietary Supplement Containing Rosemary Extract,” Journal of Clinical and Aesthetic Dermatology 18, no. 3 (2025): 34–38. PMC11932104 40135177

[15] Z. D. Draelos , A. Gueniche , M. Yatskayer , and D. B. Nelson , “A Single‐Center, Double‐Blinded, Randomized, Placebo‐Controlled Trial Evaluating the Safety and Efficacy of a Dietary Supplement Containing Rosemary Extract on Visible Facial Skin Quality,” Journal of Clinical and Aesthetic Dermatology 18, no. 3 (2025): 28–33. PMC11932106 40135175

[16] Y. Kimura , M. Sumiyoshi , T. Kobayashi , “Whey Peptides Prevent Chronic Ultraviolet B Radiation‐Induced Skin Aging in Melanin‐Possessing Male Hairless Mice,” Journal of Nutrition 144, no. 1 (2014): 27–32, 10.3945/jn.113.180406. 24174624

[17] M. Igase , Y. Okada , K. Igase , et al., “Casein Hydrolysate Containing Milk‐Derived Peptides Reduces Facial Pigmentation Partly by Decreasing Advanced Glycation End Products in the Skin: A Randomized Double‐Blind Placebo‐Controlled Trial,” Rejuvenation Research 24, no. 2 (2021): 97–103, 10.1089/rej.2020.2343. 32829654

[18] R. Meerwaldt , R. Graaff , P. H. N. Oomen , et al., “Simple Non‐Invasive Assessment of Advanced Glycation Endproduct Accumulation,” Diabetologia 47, no. 7 (2004): 1324–1330, 10.1007/s00125-004-1451-2. 15243705

[19] Y. Doi , M. Minowa , M. Uchiyama , et al., “Psychometric Assessment of Subjective Sleep Quality Using the Japanese Version of the Pittsburgh Sleep Quality Index (PSQI‐J) in Psychiatric Disordered and Control Subjects,” Psychiatry Research 97, no. 2–3 (2000): 165–172, 10.1016/S0165-1781(00)00232-8. 11166088

[20] Y. Doi , M. Minowa , M. Okawa , and M. Uchiyama , “Development of the Japanese Version of the Pittsburgh Sleep Quality Index,” Japanese Journal of Psychiatry Treatment 13, no. 6 (1998): 755–763.

[21] D. J. Buysse , C. F. Reynolds , T. H. Monk , S. R. Berman , and D. J. Kupfer , “The Pittsburgh Sleep Quality Index: A New Instrument for Psychiatric Practice and Research,” Psychiatry Research 28, no. 2 (1989): 193–213, 10.1016/0165-1781(89)90047-4. 2748771

[22] S. Endo , H. Mitsuzumi , S. Uchida , et al., “Dietary Glucosyl Hesperidin Improves Skin Color and Skin Conditions in Women,” Japanese Pharmacology & Therapeutics 43, no. 12 (2015): 1687–1699.

[23] E. Aafi , M. R. Shams Ardakani , S. Ahmad Nasrollahi , et al., “Brightening Effect of *Ziziphus jujuba* (Jujube) Fruit Extract on Facial Skin: A Randomized, Double‐Blind, Clinical Study,” Dermatologic Therapy 35, no. 7 (2022): e15535, 10.1111/dth.15535. 35460145

[24] K. Shoji , A. Kameda , and K. Furuich , “Effects of Milk Amazake on Skin Elasticity, Hydration, and Transepidermal Water Loss: An 8‐Week Double‐Blind, Randomized, Controlled Trial,” Journal of Oleo Science 72, no. 3 (2023): 329–335, 10.5650/jos.ess22342. 36878586

[25] F. Faul , E. Erdfelder , A. Buchner , and A. G. Lang , “Statistical Power Analyses Using G*Power 3.1: Tests for Correlation and Regression Analyses,” Behavior Research Methods 41, no. 4 (2009): 1149–1160, 10.3758/BRM.41.4.1149. 19897823

[26] M. Koetsier , H. L. Lutgers , C. de Jonge , T. P. Links , A. J. Smit , and R. Graaff , “Reference Values of Skin Autofluorescence,” Diabetes Technology & Therapeutics 12, no. 5 (2010): 399–403, 10.1089/dia.2009.0113. 20388050

[27] A. Goldsberry , C. W. Hanke , and K. E. Hanke , “VISIA System: A Possible Tool in the Cosmetic Practice,” Journal of Drugs in Dermatology 13, no. 11 (2014): 1312–1314. 25607694

[28] D. Martinovic , D. Tokic , M. Usljebrka , et al., “The Association Between the Level of Advanced Glycation End Products and Objective Skin Quality Parameters,” Life 13, no. 2 (2023): 256, 10.3390/life13020256. 36836618 PMC9961659

[29] J. Fang , M. Ouyang , Y. Qu , et al., “Advanced Glycation End Products Promote Melanogenesis by Activating NLRP3 Inflammasome in Human Dermal Fibroblasts,” Journal of Investigative Dermatology 142, no. 10 (2022): 2591–2602.e8, 10.1016/j.jid.2022.03.025. 35421403

[30] E. J. Lee , J. Y. Kim , and S. H. Oh , “Advanced Glycation End Products (AGEs) Promote Melanogenesis Through Receptor for AGEs,” Scientific Reports 13, no. 6 (2016): 27848, 10.1038/srep27848. PMC4904211 27293210

[31] E. Yoshinaga , A. Kawada , and K. Ono , “N(ɛ)‐(Carboxymethyl)lysine Modification of Elastin Alters Its Biological Properties: Implications for the Accumulation of Abnormal Elastic Fibers in Actinic Elastosis,” Journal of Investigative Dermatology 132, no. 2 (2012): 315–323, 10.1038/jid.2011.298. 21956123

[32] S. Konishi , S. Hatakeyama , and A. Imai , “Effect of Advanced Glycation End Products on Nocturia or Sleep Disorders: A Longitudinal Study,” BJUI Compass 3, no. 2 (2021): 162–168, 10.1002/bco2.114. 35474730 PMC8988819

[33] GenomeAsia100K Consortium , “The GenomeAsia 100K Project Enables Genetic Discoveries Across Asia,” Nature 576 (2019): 106–111, 10.1038/s41586-019-1793-z. 31802016 PMC7054211

