# Supplementary material for: Effects of Milk Protein Hydrolysates on Skin Quality and Advanced Glycation End Products in Women With Perceived Skin Dullness: Two 8‐Week Randomized Controlled Trials
Source: J Cosmet Dermatol. 2026 Jul 10;25(7):e71032. doi: 10.1111/jocd.71032 (PMC13354885; doi:10.1111/jocd.71032)
Supplement: Supplementary file 1 — Table S1: Assessment of facial swelling, fatigue, cold hypersensitivity in the hands and feet, and hair loss by VAS in Trial 1. Table S2: Assessment of skin quality by VISIA in Trial 1. Table S3: Assessment of skin quality by dermatologists in Trial 1. Table S4: Assessment of glycation stress marker and skin blood flow in Trial 1. Table S5: Assessment of sleep quality by PSQI‐J in Trial 1. Table S6: Assessment of skin quality by the spectrophotometer in Trial 2. Table S7: Assessment of skin quality by dermatologists in Trial 2. Table S8: Assessment of skin quality by VAS in Trial 2. Table S9: Assessment of sleep quality by PSQI‐J in trial 2. [file JOCD-25-e71032-s001.docx]

**Table S1.** Assessment of facial swelling, fatigue, cold hypersensitivity in the hands and feet, and hair loss by VAS in Trial 1

|  | **Group** | **Baseline** | **Week 4** | **Week 8** |
| --- | --- | --- | --- | --- |
| Facial swelling | MMP1C  MMP2W  Placebo | 64.6 ± 15.4  60.4 ± 16.1  53.9 ± 21.3 | 48.3 ± 13.6^**^  45.8 ± 15.8^**^  46.1 ± 16.1^*^ | 41.2 ± 13.2^***^  48.4 ± 17.6^*^  41.0 ± 15.7^**^ |
| Fatigue | MMP1C  MMP2W  Placebo | 64.4 ± 17.2  68.0 ± 18.8  58.8 ± 18.2 | 47.8 ± 13.0^***^  49.8 ± 16.1^**^  53.9 ± 10.7 | 42.9 ± 11.3^***^  56.0 ± 20.3^*^  47.2 ± 16.0^*^ |
| Cold hypersensitivity in the hands and feet | MMP1C  MMP2W  Placebo | 52.3 ± 21.9  52.8 ± 22.1  42.8 ± 18.0 | 51.1 ± 18.7  45.9 ± 15.9  47.0 ± 20.9 | 46.0 ± 19.9  49.5 ± 17.5  47.4 ± 18.0 |
| Hair loss | MMP1C  MMP2W  Placebo | 62.6 ± 10.7  56.5 ± 23.6  45.2 ± 21.5 | 52.3 ± 7.4^***^  51.2 ± 18.1  49.3 ± 18.9 | 45.6 ± 12.5^***^  53.5 ± 20.5  45.9 ± 16.4 |

Data represent mean ± standard deviation. The sample sizes were as follows: MMP1C group, *n =* 19; MMP2W group, *n =* 19; and placebo group, *n =* 20. ^*^*P* < 0.05, ^**^*P* < 0.01, and ^***^*P* < 0.001 compared with baseline using the paired *t-*test. There were no significant differences between the groups by ANCOVA adjusted for baseline, with Fisher’s protected least significant difference post hoc test and the closed-testing procedure. ANCOVA, analysis of covariance; VAS, visual analog scale.

**Table S2.** Assessment of skin quality by VISIA in Trial 1

|  | **Group** | **Baseline** | **Week 4** | **Week 8** |
| --- | --- | --- | --- | --- |
| Spots | MMP1C  MMP2W  Placebo | 30.2 ± 10.5  29.4 ± 6.3  31.7 ± 7.1 | 29.9 ± 9.7  28.5 ± 6.6  32.2 ± 6.7 | 30.2 ± 10.4  28.2 ± 6.4  31.7 ± 8.8 |
| Wrinkles | MMP1C  MMP2W  Placebo | 29.7 ± 22.8  26.5 ± 21.2  39.7 ± 26.3 | 29.2 ± 19.4  23.2 ± 19.6  35.4 ± 22.6 | 29.2 ± 22.7  26.5 ± 16.6  33.3 ± 19.4 |
| Texture | MMP1C  MMP2W  Placebo | 10.3 ± 5.1  9.2 ± 5.4  9.5 ± 4.2 | 10.7 ± 5.7  8.7 ± 5.0  9.1 ± 3.7 | 10.5 ± 6.0  8.7 ± 4.7  9.2 ± 3.9 |
| Pores | MMP1C  MMP2W  Placebo | 20.4 ± 10.2  21.7 ± 18.0  17.3 ± 9.4 | 21.7 ± 12.0^*^  22.3 ± 16.9  19.0 ± 10.5^*^ | 21.4 ± 11.7  22.4 ± 15.6  19.0 ± 10.0^*^ |
| UV spots | MMP1C  MMP2W  Placebo | 28.6 ± 5.1  28.1 ± 5.0  27.8 ± 6.1 | 28.7 ± 5.5  28.3 ± 5.0  28.3 ± 5.9 | 29.2 ± 5.6  29.3 ± 4.4^**^  29.0 ± 5.4^*^ |
| Brown spots | MMP1C  MMP2W  Placebo | 56.8 ± 4.8  56.8 ± 4.4  55.9 ± 6.5 | 56.7 ± 5.7  56.2 ± 5.7  56.2 ± 5.7 | 57.4 ± 4.6  56.5 ± 6.3  57.5 ± 5.4 |
| Red areas | MMP1C  MMP2W  Placebo | 34.8 ± 10.4  32.4 ± 7.2  33.0 ± 7.6 | 35.8 ± 10.8  34.6 ± 7.1  33.8 ± 7.9 | 35.2 ± 9.3  34.7 ± 7.6  35.7 ± 10.4 |
| Porphyrins | MMP1C  MMP2W  Placebo | 10.2 ± 9.7  10.2 ± 10.0  5.7 ± 4.3 | 9.7 ± 9.9  9.3 ± 8.1  5.1 ± 4.4 | 9.9 ± 9.9  9.7 ± 8.8  4.9 ± 3.1 |

Data represent mean ± standard deviation. The sample sizes were as follows: MMP1C group, *n =* 19 (week 4, *n =* 18); MMP2W group, *n =* 19; and placebo group, *n =* 20. ^*^*P* < 0.05 and ^**^*P* < 0.01 compared with baseline using the paired *t-*test. There were no significant differences between the groups by ANCOVA adjusted for baseline, with Fisher’s protected least significant difference post hoc test and the closed-testing procedure. ANCOVA, analysis of covariance; UV, ultraviolet.

**Table S3.** Assessment of skin quality by dermatologists in Trial 1

|  | **Group** | **Baseline** | **Week 4** | **Week 8** |
| --- | --- | --- | --- | --- |
| **Skin texture** |  |  |  |  |
| Skin ridges | MMP1C  MMP2W  Placebo | −0.2 ± 1.0  −0.2 ± 1.3  0.1 ± 1.2 | 0.2 ± 1.1  0.2 ± 1.0^*^  0.4 ± 1.2^*^ | 0.4 ± 1.1^*^  0.7 ± 1.0^***^  0.8 ± 1.1^***^ |
| Skin grooves | MMP1C  MMP2W  Placebo | 0.1 ± 0.8  0.1 ± 1.1  0.2 ± 1.1 | 0.6 ± 1.1^**^  0.6 ± 1.1^**^  0.6 ± 1.1^**^ | 0.7 ± 1.0^***^  0.8 ± 0.9^***^  0.9 ± 1.0^***^ |
| Comprehensive assessment | MMP1C  MMP2W  Placebo | 0.0 ± 0.9  −0.1 ± 1.1  0.1 ± 1.1 | 0.4 ± 1.1^*^  0.3 ± 0.9^**^  0.6 ± 1.2^**^ | 0.7 ± 1.0^**^  0.7 ± 0.9^***^  0.8 ± 1.1^***^ |
| **Skin quality** |  |  |  |  |
| Skin dryness | MMP1C  MMP2W  Placebo | 2.0 ± 1.3  2.6 ± 0.8  2.5 ± 0.9 | 1.9 ± 1.2  2.4 ± 0.8  2.6 ± 0.7 | 1.9 ± 1.0  2.4 ± 0.9  2.6 ± 0.7 |
| Skin erythema | MMP1C  MMP2W  Placebo | 2.7 ± 0.7  2.6 ± 0.6  2.6 ± 0.5 | 2.5 ± 0.6  2.4 ± 0.6  2.4 ± 0.5^*^ | 2.5 ± 0.7  2.4 ± 0.5  2.2 ± 0.4^**^ |
| Skin scales | MMP1C  MMP2W  Placebo | 2.2 ± 0.9  2.1 ± 0.9  2.1 ± 1.0 | 1.8 ± 0.7^*^  1.8 ± 0.9  2.0 ± 1.1 | 2.1 ± 0.8  1.9 ± 0.8  2.1 ± 0.9 |
| Skin irritation | MMP1C  MMP2W  Placebo | 0.2 ± 0.6  0.1 ± 0.5  0.2 ± 0.5 | 0.1 ± 0.5  0.1 ± 0.5  0.0 ± 0.0 | 0.1 ± 0.2  0.1 ± 0.2  0.0 ± 0.0 |
| Skin itching | MMP1C  MMP2W  Placebo | 0.2 ± 0.6  0.2 ± 0.6  0.3 ± 0.6 | 0.0 ± 0.0  0.2 ± 0.6  0.1 ± 0.2 | 0.0 ± 0.0  0.3 ± 0.7  0.0 ± 0.0 |

Data represent mean ± standard deviation. The sample sizes were as follows: MMP1C group, *n =* 19 (week 4, *n =* 18); MMP2W group, *n =* 19; and placebo group, *n =* 20. ^*^*P* < 0.05, ^**^*P* < 0.01, and ^***^*P* < 0.001 compared with baseline using the paired *t-*test. There were no significant differences between the groups by ANCOVA adjusted for baseline, with Fisher’s protected least significant difference post hoc test and the closed-testing procedure. ANCOVA, analysis of covariance.

**Table S4.** Assessment of glycation stress marker and skin blood flow in Trial 1

|  | **Group** | **Baseline** | **Week 4** | **Week 8** |
| --- | --- | --- | --- | --- |
| **Glycation stress marker** | |  |  |  |
| AGE score | MMP1C  MMP2W  Placebo | 1.99 ± 0.30  2.03 ± 0.32  2.01 ± 0.34 | 1.92 ± 0.33  2.02 ± 0.27  2.04 ± 0.35 | 1.93 ± 0.29  2.03 ± 0.32  2.03 ± 0.34 |
| **Skin blood flow** | |  |  |  |
| Number of  blood vessels | MMP1C  MMP2W  Placebo | 8.8 ± 1.4  7.9 ± 2.1  7.9 ± 2.2 | 8.5 ± 1.2  8.1 ± 2.1  8.1 ± 2.4 | 8.6 ± 1.2  8.2 ± 2.0  7.9 ± 2.2 |
| Blood flow velocity (μm/s) | MMP1C  MMP2W  Placebo | 571.5 ± 305.7  666.8 ± 450.8  712.1 ± 438.1 | 792.7 ± 427.2^*^  939.2 ± 488.0^*^ 1030.2 ± 409.2^**^ | 928.9 ± 497.3^***^  990.5 ± 394.9^**^  954.1 ± 344.9^**^ |
| Vessel diameter  [arterial side]  (μm) | MMP1C  MMP2W  Placebo | 10.1 ± 3.0  11.2 ± 2.3  10.3 ± 1.7 | 10.9 ± 2.3  12.3 ± 3.3  9.9 ± 1.7 | 10.6 ± 2.0  13.5 ± 7.1  10.9 ± 2.6 |
| Vessel diameter  [center]  (μm) | MMP1C  MMP2W  Placebo | 15.1 ± 4.2  17.1 ± 3.6  16.8 ± 4.3 | 15.7 ± 3.1  21.3 ± 6.9^**^  17.9 ± 4.7 | 16.6 ± 3.2  21.1 ± 8.1^*^  17.9 ± 4.7 |
| Vessel diameter  [venous side]  (μm) | MMP1C  MMP2W  Placebo | 12.6 ± 3.2  15.4 ± 2.8  12.4 ± 2.7 | 13.0 ± 4.5  16.5 ± 6.7  13.6 ± 3.0 | 14.2 ± 3.1^*^  18.0 ± 10.3  14.1 ± 3.6^***^ |

Data represent mean ± standard deviation. The sample sizes were as follows: MMP1C group, *n =* 19 (week 4, *n =* 18); MMP2W group, *n =* 19; and placebo group, *n =* 20. ^*^*P* < 0.05, ^**^*P* < 0.01, and ^***^*P* < 0.001 compared with baseline using the paired *t-*test. There were no significant differences between the groups by ANCOVA adjusted for baseline, with Fisher’s protected least significant difference post hoc test and the closed-testing procedure. AGE, advanced glycation end products; ANCOVA, analysis of covariance.

**Table S5.** Assessment of sleep quality by PSQI-J in Trial 1

|  | **Group** | **Baseline** | **Week 4** | **Week 8** |
| --- | --- | --- | --- | --- |
| Subjective sleep quality | MMP1C  MMP2W  Placebo | 1.3 ± 0.6  1.1 ± 0.7  1.2 ± 0.5 | 1.2 ± 0.4  1.2 ± 0.5  1.0 ± 0.5 | 1.0 ± 0.5^**^  1.3 ± 0.5  1.1 ± 0.3 |
| Sleep latency | MMP1C  MMP2W  Placebo | 1.1 ± 0.8  0.7 ± 0.8  1.1 ± 0.9 | 0.6 ± 0.8^*^  0.6 ± 0.8  0.9 ± 1.0 | 0.4 ± 0.6^***^  0.7 ± 1.0  0.7 ± 0.9 |
| Sleep duration | MMP1C  MMP2W  Placebo | 1.3 ± 0.7  1.2 ± 0.8  1.1 ± 0.8 | 1.0 ± 0.8  0.9 ± 0.9^*^  1.1 ± 0.9 | 0.9 ± 0.7^*^  1.1 ± 0.8  1.1 ± 0.8 |
| Habitual sleep efficiency | MMP1C  MMP2W  Placebo | 0.1 ± 0.3  0.1 ± 0.3  0.0 ± 0.0 | 0.0 ± 0.0  0.1 ± 0.2  0.1 ± 0.3 | 0.2 ± 0.7  0.1 ± 0.2  0.4 ± 0.9 |
| Sleep disturbances | MMP1C  MMP2W  Placebo | 0.7 ± 0.5  0.5 ± 0.6  0.8 ± 0.4 | 0.7 ± 0.5  0.7 ± 0.5  0.7 ± 0.5 | 0.5 ± 0.5  0.7 ± 0.6  0.6 ± 0.5 |
| Sleeping medications | MMP1C  MMP2W  Placebo | 0.0 ± 0.0  0.0 ± 0.0  0.0 ± 0.0 | 0.0 ± 0.0  0.0 ± 0.0  0.0 ± 0.0 | 0.0 ± 0.0  0.0 ± 0.0  0.0 ± 0.0 |
| Daytime dysfunction | MMP1C  MMP2W  Placebo | 0.4 ± 0.6  0.5 ± 0.7  0.2 ± 0.4 | 0.4 ± 0.5  0.5 ± 0.5  0.5 ± 0.6^*^ | 0.4 ± 0.7  0.5 ± 0.5  0.2 ± 0.5 |
| PSQI global score  (PSQIG) | MMP1C  MMP2W  Placebo | 4.8 ± 2.4  4.1 ± 2.4  4.2 ± 2.0 | 3.8 ± 1.9^*^  3.9 ± 2.1  4.2 ± 2.3 | 3.4 ± 1.9^**^  4.3 ± 2.4  4.1 ± 2.4 |

Data represent mean ± standard deviation. The sample sizes were as follows: MMP1C group, *n =* 19; MMP2W group, *n =* 19; and placebo group, *n =* 20. ^*^*P* < 0.05, ^**^*P* < 0.01, and ^***^*P* < 0.001 compared with baseline using the paired *t-*test. There were no significant differences between the groups by ANCOVA adjusted for baseline, with Fisher’s protected least significant difference post hoc test and the closed-testing procedure. ANCOVA, analysis of covariance; PSQI-J, Japanese version of the Pittsburgh Sleep Quality Index.

**Table S6.** Assessment of skin quality by the spectrophotometer in Trial 2

|  | **Group** | **Baseline** | **Week 4** | **Week 8** |
| --- | --- | --- | --- | --- |
| L* | MMP1C  Placebo | 65.23 ± 3.02  65.59 ± 2.40 | 65.25 ± 3.41  65.57 ± 2.06 | 65.15 ± 3.08  65.68 ± 2.33 |
| a* | MMP1C Placebo | 9.42 ± 1.61  9.04 ± 1.38 | 9.81 ± 2.07^*^  9.53 ± 1.32^***^ | 10.02 ± 1.90^***^  9.61 ± 1.43^***^ |
| b* | MMP1C Placebo | 17.34 ± 1.77  17.60 ± 1.50 | 16.94 ± 1.95^**^  17.32 ± 1.76^*^ | 16.83 ± 1.95^**^  17.06 ± 1.85^***^ |
| Melanin index | MMP1C Placebo | 1.08 ± 0.14  1.06 ± 0.13 | 1.06 ± 0.16^*^  1.05 ± 0.14 | 1.05 ± 0.16^*^  1.04 ± 0.14^**^ |
| Hb index | MMP1C Placebo | 1.30 ± 0.37  1.25 ± 0.27 | 1.37 ± 0.43^*^  1.33 ± 0.24^**^ | 1.43 ± 0.40^***^  1.36 ± 0.30^***^ |
| Hb SO2 index  (%) | MMP1C  Placebo | 52.28 ± 6.10  51.59 ± 5.59 | 54.59 ± 5.56^***^  54.78 ± 5.22^***^ | 55.11 ± 4.74^***^  54.81 ± 5.27^***^ |

Data represent mean ± standard deviation. The sample sizes were as follows: MMP1C group, *n =* 48; and placebo group, *n =* 50 (week 4, *n =* 49). ^*^*P* < 0.05, ^**^*P* < 0.01, and ^***^*P* < 0.001 compared with baseline using the paired *t-*test. There were no significant differences between the groups by ANCOVA adjusted for baseline and the closed-testing procedure. a*, degree of redness; ANCOVA, analysis of covariance; b*, degree of yellowness; Hb, hemoglobin; L*, skin lightness; SO2, oxygen saturation.

**Table S7.** Assessment of skin quality by dermatologists in Trial 2

|  | **Group** | **Baseline** | **Week 4** | **Week 8** |
| --- | --- | --- | --- | --- |
| **Skin texture** |  |  |  |  |
| Skin ridges | MMP1C  Placebo | −0.2 ± 1.2  −0.3 ± 1.2 | 0.1 ± 1.1^**^  0.1 ± 1.0^***^ | 0.2 ± 1.0^**^  0.3 ± 1.0^***^ |
| Skin grooves | MMP1C  Placebo | 0.0 ± 1.2  0.0 ± 1.1 | 0.5 ± 1.0^***^  0.5 ± 1.0^***^ | 0.7 ± 1.0^***^  0.8 ± 1.0^***^ |
| Comprehensive assessment | MMP1C  Placebo | 0.0 ± 1.2  −0.2 ± 1.2 | 0.3 ± 1.0^**^  0.3 ± 0.9^***^ | 0.5 ± 1.0^***^  0.6 ± 1.0^***^ |
| **Skin quality** |  |  |  |  |
| Skin dryness | MMP1C  Placebo | 2.5 ± 0.9  2.7 ± 1.0 | 2.7 ± 1.0  2.7 ± 1.0 | 2.7 ± 1.0  2.6 ± 1.0 |
| Skin erythema | MMP1C  Placebo | 3.2 ± 0.7  3.0 ± 0.7 | 3.1 ± 0.7  2.9 ± 0.6^*^ | 3.1 ± 0.7  3.0 ± 0.7 |
| Skin scales | MMP1C  Placebo | 2.5 ± 1.2  2.7 ± 1.1 | 2.4 ± 0.9  2.5 ± 0.9 | 2.2 ± 1.1  2.3 ± 1.0^**^ |
| Skin irritation | MMP1C  Placebo | 0.2 ± 0.7  0.0 ± 0.3 | 0.2 ± 0.6  0.2 ± 0.8 | 0.1 ± 0.4  0.1 ± 0.4 |
| Skin itching | MMP1C  Placebo | 0.1 ± 0.4  0.2 ± 0.7 | 0.1 ± 0.4  0.1 ± 0.4 | 0.1 ± 0.4  0.1 ± 0.4 |

Data represent mean ± standard deviation. The sample sizes were as follows: MMP1C group, *n =* 48; and placebo group, *n =* 50 (week 4, *n =* 49). ^*^*P* < 0.05, ^**^*P* < 0.01, and ^***^*P* < 0.001 compared with baseline using the paired *t-*test. There were no significant differences between the groups by ANCOVA adjusted for baseline and the closed-testing procedure. ANCOVA, analysis of covariance.

**Table S8.** Assessment of skin quality by VAS in Trial 2

|  | **Group** | **Baseline** | **Week 4** | **Week 8** |
| --- | --- | --- | --- | --- |
| Skin dullness | MMP1C  Placebo | 68.0 ± 11.2  69.2 ± 11.2 | 52.1 ± 10.8^***^  51.1 ± 13.6^***^ | 48.3 ± 12.3^***^  48.0 ± 15.4^***^ |
| Skin spots | MMP1C  Placebo | 71.1 ± 11.4  71.6 ± 12.5 | 56.0 ± 11.3^***^  56.8 ± 14.1^***^ | 51.8 ± 12.1^***^  53.4 ± 14.3^***^ |
| Skin texture | MMP1C  Placebo | 67.1 ± 12.7  66.2 ± 12.8 | 52.4 ± 10.6^***^  54.1 ± 15.0^***^ | 49.6 ± 14.8^***^  49.4 ± 15.5^***^ |
| Skin elasticity | MMP1C  Placebo | 66.6 ± 11.9  66.0 ± 15.0 | 52.3 ± 12.4^***^  54.5 ± 14.3^***^ | 51.0 ± 14.2^***^  50.9 ± 16.3^***^ |
| Skin gloss | MMP1C  Placebo | 66.5 ± 12.0  63.8 ± 14.8 | 52.4 ± 12.2^***^  51.8 ± 15.9^***^ | 51.6 ± 15.5^***^  49.8 ± 14.9^***^ |
| Skin clarity | MMP1C  Placebo | 70.3 ± 11.8  69.5 ± 12.9 | 53.3 ± 10.7^***^  54.7 ± 13.0^***^ | 51.0 ± 13.4^***^  50.1 ± 15.4^***^ |
| Skin redness | MMP1C  Placebo | 54.9 ± 15.4  51.8 ± 17.0 | 48.7 ± 16.6^*^  45.5 ± 17.2^*^ | 47.1 ± 14.9^**^  43.2 ± 16.2^**^ |
| Skin brightness | MMP1C  Placebo | 65.1 ± 11.8  65.3 ± 12.3 | 51.2 ± 12.4^***^  49.1 ± 14.9^***^ | 48.2 ± 13.9^***^  46.7 ± 15.7^***^ |
| Skin moisture | MMP1C  Placebo | 65.6 ± 12.1  64.1 ± 14.8 | 52.8 ± 13.6^***^  52.9 ± 17.3^***^ | 50.9 ± 14.9^***^  49.0 ± 18.0^***^ |
| Makeup adhesion | MMP1C  Placebo | 63.8 ± 12.9  62.6 ± 13.4 | 53.1 ± 12.5^***^  51.3 ± 17.7^***^ | 49.1 ± 15.1^***^  47.8 ± 18.5^***^ |

Data represent mean ± standard deviation. The sample sizes were as follows: MMP1C group, *n =* 48; and placebo group, *n =* 50 (week 4, *n =* 49). ^*^*P* < 0.05, ^**^*P* < 0.01, and ^***^*P* < 0.001 compared with baseline using the paired *t-*test. There were no significant differences between the groups by ANCOVA adjusted for baseline and the closed-testing procedure. ANCOVA, analysis of covariance; VAS, visual analog scale.

**Table S9.** Assessment of sleep quality by PSQI-J in trial 2

|  | **Group** | **Baseline** | **Week 4** | **Week 8** |
| --- | --- | --- | --- | --- |
| Subjective sleep quality | MMP1C  Placebo | 1.1 ± 0.4  1.2 ± 0.6 | 1.1 ± 0.5  1.1 ± 0.5 | 1.0 ± 0.5  1.2 ± 0.7 |
| Sleep latency | MMP1C  Placebo | 0.6 ± 0.7  0.8 ± 0.9 | 0.7 ± 0.8  0.8 ± 0.9 | 0.7 ± 0.7  0.6 ± 0.8^*^ |
| Sleep duration | MMP1C  Placebo | 0.9 ± 0.6  1.0 ± 0.6 | 0.9 ± 0.6  0.9 ± 0.7 | 0.8 ± 0.7  1.0 ± 0.7 |
| Habitual sleep efficiency | MMP1C  Placebo | 0.0 ± 0.1  0.0 ± 0.2 | 0.1 ± 0.3  0.0 ± 0.1 | 0.0 ± 0.1  0.1 ± 0.2 |
| Sleep disturbances | MMP1C  Placebo | 0.7 ± 0.5  0.6 ± 0.5 | 0.6 ± 0.5  0.7 ± 0.5 | 0.6 ± 0.5  0.6 ± 0.5 |
| Sleeping medications | MMP1C  Placebo | 0.0 ± 0.0  0.0 ± 0.0 | 0.0 ± 0.0  0.0 ± 0.0 | 0.0 ± 0.0  0.0 ± 0.0 |
| Daytime dysfunction | MMP1C  Placebo | 0.4 ± 0.5  0.4 ± 0.6 | 0.3 ± 0.6  0.3 ± 0.6 | 0.3 ± 0.5  0.4 ± 0.6 |
| PSQI global score  (PSQIG) | MMP1C  Placebo | 3.7 ± 1.6  3.9 ± 2.2 | 3.7 ± 1.9  3.9 ± 2.3 | 3.4 ± 1.7  3.8 ± 2.3 |

Data represent mean ± standard deviation. The sample sizes were as follows: MMP1C group, *n =* 48; and placebo group, *n =* 50 (week 4, *n =* 49). ^*^*P* < 0.05 compared with baseline using the paired *t-*test. There were no significant differences between the groups by ANCOVA adjusted for baseline and the closed-testing procedure. ANCOVA, analysis of covariance; PSQI-J, Japanese version of the Pittsburgh Sleep Quality Index.
